# Mapping Women's Community Sport Participation to Inform Sport Development Initiatives: A Case Study of Row Ontario

**DOI:** 10.3389/fspor.2022.836525

**Published:** 2022-04-05

**Authors:** Kyle A. Rich, Emily Moore, Jeffrey Boggs, Ann Pegoraro

**Affiliations:** ^1^Department of Recreation and Leisure Studies, Brock University, St. Catharines, ON, Canada; ^2^Lang School of Business and Economics, University of Guelph, Guelph, ON, Canada; ^3^Department of Geography and Tourism Studies, Brock University, St. Catharines, ON, Canada; ^4^School of Hospitality, Food and Tourism, University of Guelph, Guelph, ON, Canada

**Keywords:** sport development, gender, rural, geography, spatial analysis, case study

## Abstract

Using a spatial analysis methodology, we analyzed sport participation through membership data of a Provincial Sport Organization (PSO) in Ontario, Canada. Specifically, our analysis brings attention to the participation of women and girls in Row Ontario and the urban and rural nature of the PSO's membership. This research was conducted in partnership with the PSO to provide insights into how contextual factors influence sport participation and how these findings can inform future sport development efforts. Our results demonstrate that women and girls represented the majority of participants within the PSO and highlight an opportunity to use participant centered approaches for sport development to grow women and girls' participation. This is a promising outcome as women and girls are generally underrepresented in sport and may face greater participation barriers in rural contexts.

## Introduction

There is a current deficit in research exploring the regional differences in sport policy implementation and their subsequent outcomes. This lack of research leaves unanswered questions related to sport participation patterns across diverse community contexts. As sport participation is impacted by the social, cultural, and demographic conditions of a region, greater insights into these factors will assist sport organizations in understanding the populations they serve (Rich and Misener, 2020). In this research, we investigated the sport participation outcomes of women in Ontario, Canada. Specifically, we sought to pilot a method of spatial analysis. This work analyzed the spatial characteristics of sport participation outcomes by examining urban and rural characteristics of participants' residences. As there is no universally accepted definition of rural, we draw from the work of Bollman and Reimer (2018) to spatially analyze sport participation (using membership data). We analyzed participant demographic data from the membership of Row Ontario—one of Ontario's Provincial Sport Organizations (PSO)—and Statistics Canada's Census Subdivisions (CSDs) data to complete our analysis. In this paper, we review the methodology used to conduct this analysis and we discuss the implications it has for sport development practice.

Broadly, we sought to understand how women and girls in diverse community contexts access sport participation opportunities. To do this, we explored the nature of rural and urban membership within the PSO to identify what patterns are revealed across the geography of participant data. We first combined spatial (rural or urban location) and demographic (gender and age) data to analyze the participation of women and girls in the sport. Next, we connected these spatial and demographic patterns in women and girls' sport participation to sport development efforts within the PSO. In doing so, we generated a discussion of the regional differences in sport participation and how these differences may be accounted for in sport development efforts. While spatial analysis techniques are common in fields of health and demography (Australian Department of Health Aged Care, 2001; Australian Bureau of Statistics, 2016; Statistics Canada, 2022), they have only recently been applied in in the context of sport participation using data from sport clubs (Eime et al., 2016). For example, Eime et al. (2021) analyzed participation in 10 sports across, age, gender, and region in Victoria, Australia. Our analysis introduces this methodological approach to examine participation in rowing in Ontario, Canada. The analysis documented here highlights how regional differences are evident, and provides a foundation upon which further examination of sport policy implementation in Ontario can be examined. These outcomes can inform strategic efforts aimed at increasing women and girl's sport participation. We review relevant literature before elaborating on our methodology and discussing its implications for research and practice.

### Women and Girl's Sport Participation in Canada

Canadian women's involvement in sport has increased in recent years (Government of Canada, 2009). Today, Canadian women have greater representation in sport administration, and as athletes at the Olympic and Paralympic games (Government of Canada, 2009). At the 2020 Olympic games over half of Canada's athletes were women (60%) and 55% of Canada's Paralympic team were women athletes (Canadian Olympic Committee, 2021a; Canadian Paralympic Committee, 2021a). Further, these athlete's won the majority of Canada's medals, with 75% of the Olympic and 67% of the Paralympic medals (Canadian Olympic Committee, 2021b; Canadian Paralympic Committee, 2021b). However, despite these encouraging changes, women remain under-represented in sport participation and leadership. Men still make up the majority of professional coaches in university and college sports (Canadian Women Sport, 2020a). Most women and girls are not involved in sport programs, as 62% of Canadian girls and <20% of Canadian women aged 16–63 participate in sport (Canadian Women Sport, 2020b). These statistics are the result of high sport dropout rates, as one in three girls leave sport in adolescence, compared to one in 10 boys (Canadian Women Sport, 2020b). As a result, girls and women have lower sport participation rates later in life, as men continue to participate at a higher rate across their lifetime (Canadian Women Sport, 2020b). For instance, 25% of men aged 55–63 participate in sport, in comparison to 13% of women (Canadian Women Sport, 2020b). Further, for men between the ages of 24–31, 43% participate in sport, which is more than double that of the 21% of women of the same age bracket (Canadian Women Sport, 2020b). These inequities are echoed at the administrative level, as men fill the majority of coaching roles for high performance, university, and college teams (Government of Canada, 2009; Canadian Women Sport, 2020b). Further, most Canadian community sport volunteers are men, who fill 64% of these positions (Doherty, 2005). The exclusion of Canadian women and girls from sport, is cause for concern, as the voices of these populations remain absent from the world of sport participation and administration, and these individuals are not receiving the benefits that may be realized through sport participation. These benefits include enhanced mental health, improved physical fitness and improved social connections (Canadian Centre for Ethics in Sport CCES, 2022).

In response to these concerns, there has been a sustained effort to increase women and girls' participation in Canadian sport through policy intervention. In 1986 the Federal government published Sport Canada's Policy on Women in Sport, outlining efforts to reduce sport gender segregation (Government of Canada, 2009). As a result, Sport Canada now reserves funding for organizations who have formalized commitments for women's participation (Canadian Women Sport, 2020b). The current policy, *Actively Engaged: A Policy on Sport for Women and Girls*, published in 2009 sets objectives to foster sport environments where women and girls receive quality sport experiences and equitable support (Canadian Women Sport, 2020b).

The attainment of these quality sport experiences is contingent on women and girl's increased sport participation. Existing outdated societal attitudes (Hanlon et al., 2019; Canadian Women Sport, 2020b), structural issues (e.g., programming availability or funding) and individual factors such as, perceived ability and interest (Hanlon et al., 2019) are all factors which may limit women and girls' participation. These factors may be further compounded for certain populations, including individuals with disabilities or a lower socio-economic status (Government of Canada, 2009). Additional policy initiatives such as the Working Group on Gender Equity in Sport also attempt to foster meaningful sport opportunities for all girls and women in Canada (Government of Canada, 2009, 2019).

Location may also be considered a barrier in gaining access to sport. Researchers have highlighted the distinct challenges of rural communities which may have fewer sport opportunities due to their distance from higher density areas, lower population, and economic climate (Mair et al., 2019). In addition, the sport communities in rural areas may also be exclusionary, or tightly knit, posing increased barriers or constraints to participation for different demographics (Tonts and Atherly, 2010).

### Rural Women and Sport Participation

Women living in rural settings may experience intensified barriers to sport participation and leadership opportunities due to the limited resources, as well as the physical and social contexts of their communities (Warner-Smith and Brown, 2002; Smith et al., 2008). Issues of job security, access to transportation, familial commitments, and isolation have been identified as factors limiting rural women's involvement in sport (Smith et al., 2008). The notion of remoteness, or living “out-of-town” is a particularly strong barrier for girl's sport participation, as they rely on their parents for transportation to sport opportunities (Casey et al., 2009; Eime et al., 2010). Notably, rural women are often responsible for a greater workload than that of women living in urban areas, as they may have additional economic and community responsibilities (Coward et al., 2006). For example, Trussell and Shaw (2007) described farm women's experiences as “single parenthood in the marital context” given the work-related pressures of a farm household. Therefore, effective sport development efforts should reflect the nuances of rural women's lived experiences.

Research investigating rural women's community sport experiences in Southwestern Ontario suggested that women who participated in sport experienced benefits of increased social contact and improved mental and physical health, enhanced self-esteem, and a newfound interest in sport (Leipert et al., 2011). Other work has demonstrated that a participant-centered approach to rural sport development may be best suited to creating successful participation opportunities for women, allowing more women to gain access to sport and its benefits (Rich et al., 2019). This bottom-up approach can facilitate programming aligned with the context of the target demographic, which for rural women may include access to childcare, a desire for non-competitive sport, and flexible participation commitments (Rich et al., 2019). A bottom-up model challenges typical athlete development frameworks, wherein a small group of elite athletes are supported by a larger group of recreational participants, a model which does not lend itself to development within community contexts (Green, 2005; Rowe et al., 2016).

Given that sport development encapsulates processes of creating sport participation opportunities and athletic excellence as well as the promotion of other non-sport outcomes (e.g., social inclusion, cohesion, and physical literacy), contextual factors must be considered in these processes. These factors are particularly relevant for sport policy implementation intended to advance sport development in rural areas where location or context may have important impacts on sport participation outcomes. For many sport organizations, both sport participation and social outcomes are paramount, making participant driven or bottom-up approaches a relevant option (Rich and Misener, 2019). Our study addresses a research gap by applying spatial analysis methodology which considers community context in conjunction with demographic variables to identify patterns related to sport participation in Ontario, Canada.

## Methodology

Our study was informed by an instrumental case study methodology (Stake, 1995). We sought to pilot the current methodological approach to gain better understanding of the contributions that spatial analysis may make to sport development and policy theory and practice. The case study research was undertaken in partnership with stakeholders from Row Ontario, who were embedded in the research process. As such, the development of the research methodology reflected attempts to embed principals of participatory action research (Spaaij et al., 2018; Rich and Misener, 2020). This was achieved by including stakeholders from the PSO in discussions of the research design, analysis of research results, as well as the development of output for both academic and practitioner audiences (Rich et al., 2020, 2021).

Data were collected and analyzed in two phases. First, membership data from Row Ontario were collected. These data included the date-of-birth, gender, and address (including postal code) for all participants registered with the PSO from 2014 to 2019 inclusively (i.e., over the course of six seasons). To provide a broad picture of participant demographics, the participant data were coded for age and gender, and “geo-coded” using participant postal codes (using centroids of postal codes provided).

Membership data were mapped and visually depicted to examine regional differences. In collaboration with stakeholders at the PSO, we developed eight functional regions in the province. These regions were identified by the PSO based on clusters of clubs in similar geographic regions. While these regions were largely determined based on feedback from the PSO, their borders were defined using CSDs to allow for the subsequent analysis. Usually, CSD boundaries are identical to municipal boundaries (Statistics Canada, 2016, 2020). In 2016, Ontario had 575 CSDs^1^.

Next, ArcGIS software was used to locate and map sport clubs and their participants. The resulting maps locate clubs and participants within each of the identified regions. The resulting maps also allowed us to analyze spatial patterns in participation related to population distributions from Statistic Canada and the demographics (e.g., age and gender) of participants.

In the second phase, we sought to systematically examine the characteristics of the community contexts of participants. That is, we examined patterns related to urban and rural characteristics of participants' places of residence. Importantly, there is no universal definition for “rural.” Rather, rurality is characterized based on available measures, such as population (density), distances to large centers, and socio-economic characteristics. Using the work of Bollman and Reimer (2018) to guide this analysis, rurality was characterized based on population and commuting practices.

Statistic's Canada's CSD data were used (Statistics Canada, 2016, 2020), following Bollman and Reimer (2018), to produce two measures of rurality. Using 2016 population data, CSDs were categorized as low population (≤ 10,000 inhabitants) or high population (>10,000 inhabitants). The population threshold of 10,000 or less provides a useful proxy for the size of a CSD's local economy and density (Bollman and Reimer, 2018), as smaller CSDs usually have less specialized economies. CSD's were also categorized based on the number of working residents who commute outside of the CSD for employment. We used a 50% threshold (of the overall population) to categorize low and high rates of commuting for employment. This measure indicates the availability of local employment and the attraction that a population feels to another location for work. Importantly, this measure captures the remoteness of municipalities as small populations do not necessarily mean that residents are unable to access employment (and services) at nearby locations (e.g., a small bedroom community located close to an urban center). Using these two measures, we constructed a four-case typology across the two measures of rurality (Table 1).

**Table 1:** CSD identification typology.

		**Population**	
		**High (>10,000)**	**Low (≤10,000)**
Commuting rates	High (>50%)	Case 1	Case 2
	Low (≤ 50%)	Case 3	Case 4

Using these measures, each of the 575 Ontario CSDs were assigned to one of the four cases. CSDs assigned as Case 4 (i.e., low-low) are those that are considered as the most rural, as they have populations of 10,000 or lower and less than half of their working residents commuting to other CSDs for employment. At the other extreme, CSDs assigned as Case 1 are those that are considered least rural. Cases 2 and 3 indicate CSDs representing two different types of community, with intermediate levels of rurality based on one or other of the criteria. For example, a small bedroom community located close to an urban center (i.e., Case 2: low-high) may represent a very different community context than an isolated CSD with a medium-sized population (i.e., Case 3: high-low).

Finally, we sought to quantitatively assess participation patterns across the regions by examining participant age, gender, and characteristics of their community context (i.e., CSD Case). Following these phases of data collection and analysis, we had a robust depiction of sport participation within the PSO over the course of six seasons. The maps and descriptive statistics provided were then discussed with stakeholders at the PSO to identify implications for sport development and in particular, to identify implications for women and girl's participation in Row Ontario.

## Results

The following results were selected from the larger data set. In line with the participatory principals that underpinned the research project, this selection was made in collaboration with our research partners at Row Ontario. As such, the following are examples of results that were identified as most relevant for sport development practice at the PSO. Results are presented as three themes: functional regions; broader participation trends and patterns, and; participation of women and girls.

### Functional Regions

**Figure 1:**
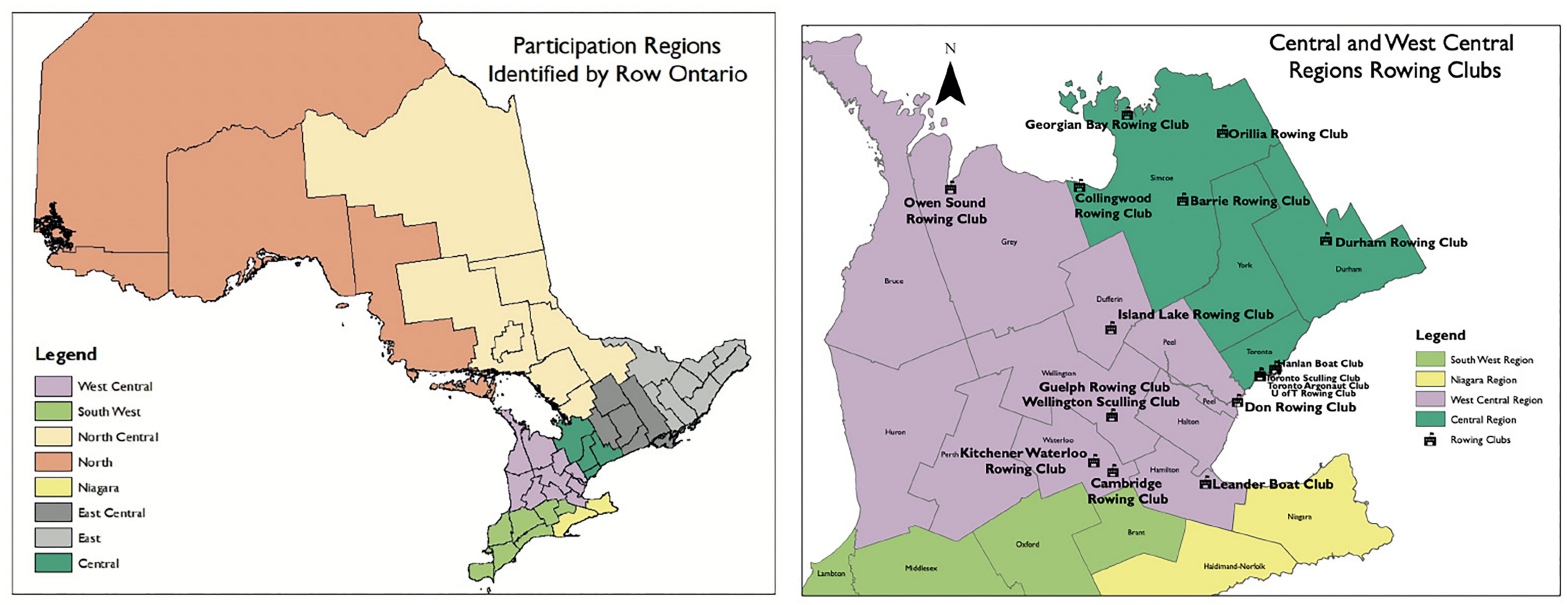
Functional regions and example of club maps for central and west central region.

Our analysis resulted in the construction of a map depicting the eight functional regions within Ontario. These functional regions, depicted in Figure 1 were constructed using both PSO clusters and CSD boundaries. The eight regions include: West Central, South West, North Central, North, Niagara, East Central, East, and Central.

The locations of rowing clubs were then mapped within the eight functional regions (see Figure 1). The overlay of these locations demonstrated the greatest density of club locations fell within the four regions of South West, Niagara, West Central, and Central. Over the 6 years of the study period, there were 59 clubs in operation and the 2019–2020 season, the most recent season in the dataset, had the highest number of clubs in operation at 53.

### Participation Trends and Patterns^2^

Across the six seasons, the total PSO membership fluctuated, peaking in the 2016–2017 season. The average number of annual participants was 6,401. The average number of participants decreased by 17% from the peak in the 2016–2017 season to the 2019–2020 season. The Niagara region had the largest average number of participants at 1,689 participants, across the 6 years of study, followed by the Central, West-Central and East regions. The North and North-Central regions had the lowest average participant range, with 77 and 178 average participants, respectively.

When the participation patterns across the province were assessed, differences in mean participant age across regions were identified. The greatest share of members were teenagers, with the largest number of participants in the 15–19 year-old cohort followed by the 10–14 year-old cohort. The general age structure across 5-year age cohorts remained stable throughout the period of study. The Niagara region maintained the youngest participants with a mean age of 20.9 (s ± 12.9) years in the 2014–2015 season, while the North region had the oldest mean participant age at 37.5 (s ± 18.1) years in the 2019–2020 season. The majority of regions had mean participants between the ages of 28 and 30. The mean participant age increased slightly from 27.3 to 29.3 years from the 2014 to 2020 seasons. The increase in mean participant age took place in all regions, except for East-Central who experienced a decrease of 1.1 years in mean participant age.

Our CSD analysis depicted that only a very small percentage of PSO members identified as rural. Only 14 members resided in Case 4 (low-low) CSD locations in the 2014–2015 season which only grew slightly to 16 in the 2019–2020 season. The members who live in these rural locations make up less than one percent of the total PSO membership. While this membership is small, it remained stable throughout our period of analysis. A similar result is observed in considering the other CSD cases, demonstrating that when participation fluctuates, membership is affected across all locations (e.g., rural or urban clubs are not appreciably affected more than one another). The majority of rural participants were 30+ years of age across the years of study. Across all years studied, there were no rural members between 23 and 29. Table 2 provides the analysis of rural participants by age group in the first and last year of the study period.

**Table 2:** Rurality of PSO members by age groups for 2014–2015 and 2019–2020 seasons.

		**2014–2015**						**2019–2020**					
	**Age**	** <15**	**15–16**	**17–18**	**19–22**	**23–29**	**30+**	** <15**	**15–16**	**17–18**	**19–22**	**23–29**	**30+**
Rural participants	*n*	1	0	1	3	0	10	1	3	1	4	0	7
	%	0.14	0	0.10	0.47	0	0.51	0.15	0.27	0.11	0.54	0	0.33
Total RO members	*n*	720	1,312	1,046	636	442	1,947	678	1,121	901	744	385	2,131

### Participation of Women and Girls

In reviewing the PSO participation results with respect to gender, we found that on average women represented 60% of PSO members in any given year. There were more women and girls at clubs across all of the eight regions with proportions ranging from 59 to 73% of total membership. Most significantly, women and girl participants were nearly triple that of boys and men in the North-Central region, with an average proportion of 73% of club membership. The North region had the second highest percentage of women and girl members with 62%.

The average age of women and girls was consistently higher than boys and men across all regions. The highest average age of women participants was found in the North and North-Central regions with average participant ages of 33 years. This is consistent with the general membership patterns for those regions, as these regions also had the highest average participant age when considering all members. The mean age of women participants in the North region was highest in the 2019–2020 season at 37.7 (s ± 17.1) years, which is noticeably higher than the average age (for women) of 30.9 (s ± 18.1) years recorded in the same region for the 2014–2015 season.

In reviewing rural women's membership, Table 3 shows that the participation of girls and women remains relatively constant throughout the period of analysis (2014–2020). Further, there are consistently more rural women and girls members across the PSO's than boys and men, with the exception of the 2018–2019 season.

**Table 3:** Rurality of PSO membership by gender in all seasons.

		**2014–2015**		**2015–2016**		**2016–2017**		**2017–2018**		**2018–2019**		**2019–2020**	
	**Gender**	**F**	**M**	**F**	**M**	**F**	**M**	**F**	**M**	**F**	**M**	**F**	**M**
Rural members	*n*	9	6	9	6	9	6	8	7	8	11	9	7
	%	0.24	0.24	0.23	0.22	0.21	0.21	0.22	0.29	0.21	0.42	0.25	0.3
Total RO members	*n*	3,706	2,447	3,979	2,698	4,305	2,879	3,652	2,413	3,862	2,597	3,608	2,319

## Discussion

The application of spatial analysis techniques to participation data from one PSO in Ontario provided notable insights for sport development. For example, the construction of the eight functional regions demonstrated that each region has differing demographic groups who may have differing contextual needs. This is in line with research conducted in (Victoria) Australia that highlighted regional differences in the sport participation patterns of women and girls (Eime et al., 2021). While regional organizational structures and policies are used by a variety of sport organizations, the use of regions to examine participation patterns at a provincial level is uncommon in Ontario. Such an approach may yield a variety of theoretical and practical insights for policymakers and researchers.

First, initiatives to increase participation should be informed by contextual patterns of participation. At an aggregate level, Row Ontario has experienced a decrease in membership across the study time period while at the same time, the average age of participants across the PSO rose by 2 years. Within different regions however, large differences in mean ages illustrate the need to tailor initiatives strategically for participants who are (or are not) engaged. For example, in the North-Central region where mean age of participants is high, initiatives to raise awareness and provide first contact opportunities to younger participants may be pertinent. In the Niagara Region, where the mean age is much lower, efforts to retain more participants beyond their early twenties might be explored. These findings align with previous research, calling for sport development to adopt a more bottom-up or participant-centered approach (Rich and Misener, 2019; Rich et al., 2019).

Considering the implications of our findings, we also recognized the opportunity of the PSO to promote participation opportunities to women, particularly those who live in rural areas. Significantly, the majority of members across all identified regions were women, a stark contrast to Canadian statistics indicating that <20% of women are active in sport (Canadian Women Sport, 2020b). It appears that this PSO has a strong women's membership base which may be capitalized to further promote the sport (or others) to other women. This opportunity may be most pertinent for women living in the North-Central and North regions where women's participation is highest, at 73 and 62%, respectively. Interestingly, these regions are less populated, represent unique social and economic contexts, and are generally considered to be more rural than other parts of the province. Therefore, a better understanding of the geography of rural (and in this case, Northern) women's sport participation in Ontario—and their experiences in rowing specifically—may be useful for researchers, practitioners, and policymakers. As rural communities may lack sufficient resources to address women's health promotion (Leipert, 2010), utilizing sport participation to improve physical, social, and community health is an important opportunity. Capitalizing on successes in these regions may serve to promote wellbeing through women and girls sport participation.

Moving toward a participant-centered orientation for sport development would allow for sport organizations to better meet the needs of participants within their unique contexts. In an analysis of rural women's stand-up paddle boarding in Australia, a bottom-up or participant-centered approach allowed for organizers to reconstruct sport delivery models to work within participants' identified constraints (Rich et al., 2019). If constraints can be negotiated, an increase in participation and access to sport related benefits may be achieved. Future work should investigate the constraints to participation within different regions, and how they are being negotiated by women, girls, and sport clubs. Existing research indicates that for women, these may include childcare support, access to equipment, as well as social and financial support (Rich et al., 2019), however future inquiry may look more broadly at other social, cultural, and economic factors that may shape participation.

Importantly, physical geographies themselves may represent important constraints and enablers to sport participation in different regions. Similar to the stand-up paddle boarding example (Rich et al., 2019), specific flat water conditions are conducive to rowing participation. Regions with few natural bodies of water that can support a rowing course may require travel over longer distances to access participation opportunities. Given that distance to services is an important consideration for rural communities (Casey et al., 2009), access to sport amenities may further restrict rural participants' engagement in different sports. In this regard, analyzing sport participation regionally provides a useful strategy to understanding sport participation and strategically planning sport development initiatives in Ontario.

### Limitations and Directions for Future Research

This case study examined one PSO in one geographic region. To assess this methodology further, comparison between sports (e.g., see the work of Eime et al., 2021) and other geographic areas is necessary. Including sports that are less location dependent or those where access to facilities is greater in both urban and rural areas (e.g., soccer) would also help to assess the implications of this methodology more broadly.

A primary limitation of our study is evident in the address information recorded for PSO members. The information provided may not be current and postal codes are an imperfect measure of members' residential locations. Further, address information does not account for members' quantity or quality of sport experience. The use of CSDs to construct regions in this method provides a significant opportunity to pair this sort of regional membership-based analysis with other data sets (e.g., time-use surveys, census data, etc.) to examine how membership patterns may be associated with other demographic changes. Further, our study lacked exploration into women's intersectional sport experiences. With additional data on participants and their communities, future work may examine how intersections influence women's sport participation from a geographic perspective.

Analytically, our four-case matrix for analysis provided a useful way to categorize community context. In determining our measures and thresholds for analysis, we followed the method of Bollman and Reimer (2018) as an established protocol for using Statistics Canada data. However, the approach could be refined for more granular measures of distance and density. Specifically, our categorization produced a very small frequency of participants in the rural (i.e., low-low) category, which calls us to accept the usefulness of the comparisons made with caution. These methodological choices do however, align with common analysis in Canadian rural development research. As such, the patterns identified highlight the importance of considering how sport development efforts can better reach and serve participants in rural areas.

Our analysis ended with the 2019–2020 season. Membership data in subsequent seasons may provide important insights on how broader events and disruptions, such as the COVID-19 pandemic, influenced participation, and membership levels. Importantly, spatial analysis may provide insights regarding how disruptions are experienced differently across regions and if they have distinctly gendered impacts. Such an analysis would help to understand the impacts of location on both the stoppage and return to sport participation in the longer term. Similar analyses may also be useful for understanding the impacts of other events, such as the hosting of a multi-sport event in a region and to compare membership patterns across many sport organizations. Future work may also use spatial methods for comparative analyses across regions or policy systems.

Collectively, spatial analysis provides exciting methodological opportunities for both researchers and practitioners in sport development and policy. The methodology we have outlined offers a strategy for a more refined analysis of community context in Canada that does not homogenize all rural and urban experiences. Considering contextual factors that impact participation in different regions may inform effective development of programs and initiatives—particularly those designed to increase participation of women and girls.

## Data Availability Statement

The original contributions presented in the study are included in the article/Supplementary Material, further inquiries can be directed to the corresponding author.

## Author Contributions

KR and AP conceptualized the project, liaised with partners, and oversaw the writing and editing of the manuscript. EM reviewed literature, wrote, and edited drafts of the manuscript. JB oversaw data analysis, writing of findings section, and editing of manuscript. KR and EM finalized draft for submission. All authors contributed to the article and approved the submitted version.

## Funding

This research was funded through the Sport Information Resource Centre Researcher-Practitioner Match Grant as well as a Brock University Explore-Exchange Grant.

## Conflict of Interest

The authors declare that the research was conducted in the absence of any commercial or financial relationships that could be construed as a potential conflict of interest.

## Publisher's Note

All claims expressed in this article are solely those of the authors and do not necessarily represent those of their affiliated organizations, or those of the publisher, the editors and the reviewers. Any product that may be evaluated in this article, or claim that may be made by its manufacturer, is not guaranteed or endorsed by the publisher.

## Footnotes

^1^CSDs can be further broken into smaller Dissemination Areas or DAs; Ontario has over 20,000. Everything we show could be performed with DAs instead of CSDs. but for our purposes, CSDs were more manageable and appropriate for this instrumental case study.

^2^Due to space limitations, all data and figures cannot Be reported in this brief report. However, more details are available in the Supplementary Materials.
