# Supplementary material for: Mapping Women's Community Sport Participation to Inform Sport Development Initiatives: A Case Study of Row Ontario
Source: Front Sports Act Living. 2022 Apr 5;4:836525. doi: 10.3389/fspor.2022.836525 (PMC9030508; doi:10.3389/fspor.2022.836525)
Supplement: Supplementary file 1 [file Data_Sheet_1.pdf]

Supplementary Data Files

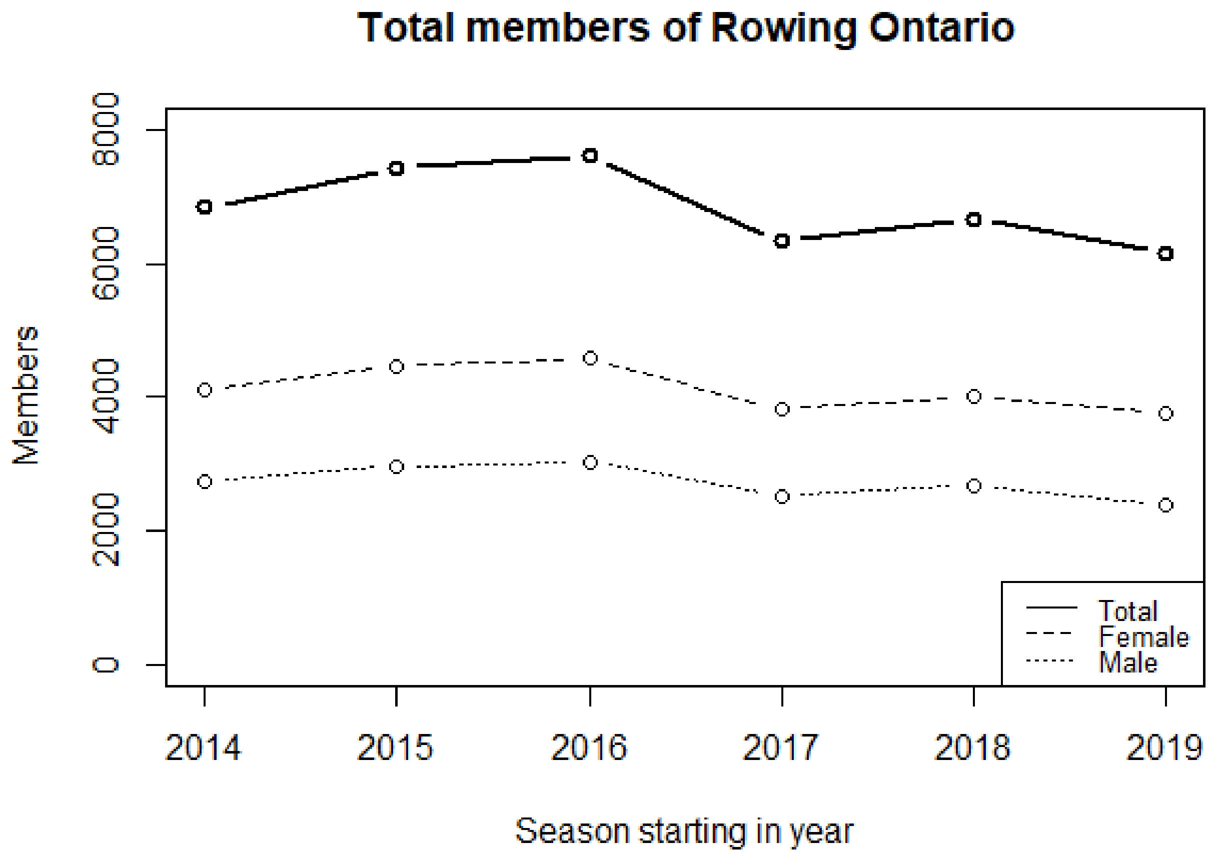

Total Row Ontario Members 2014-2019

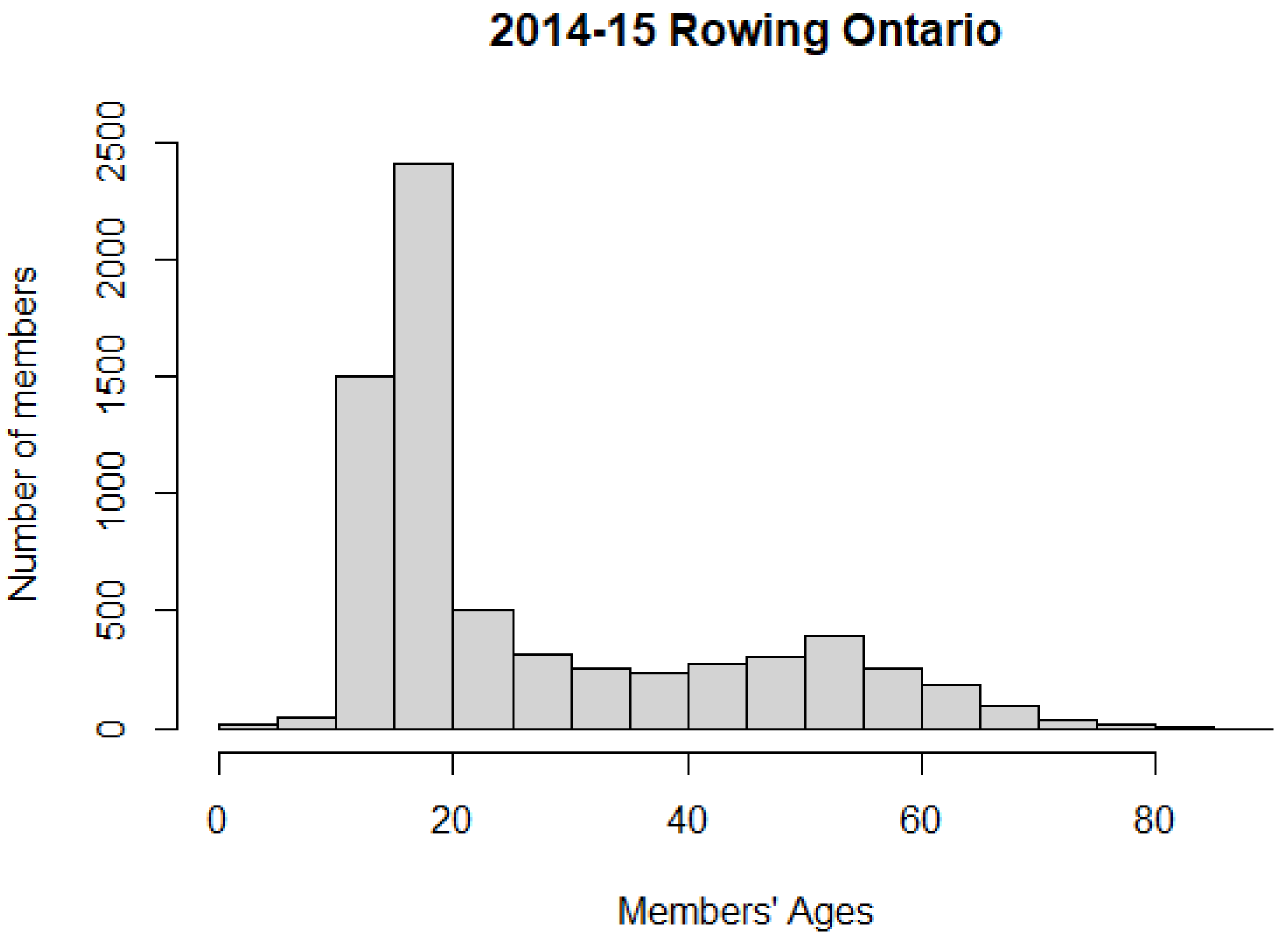

Age structure of all Row Ontario Members, 2014.

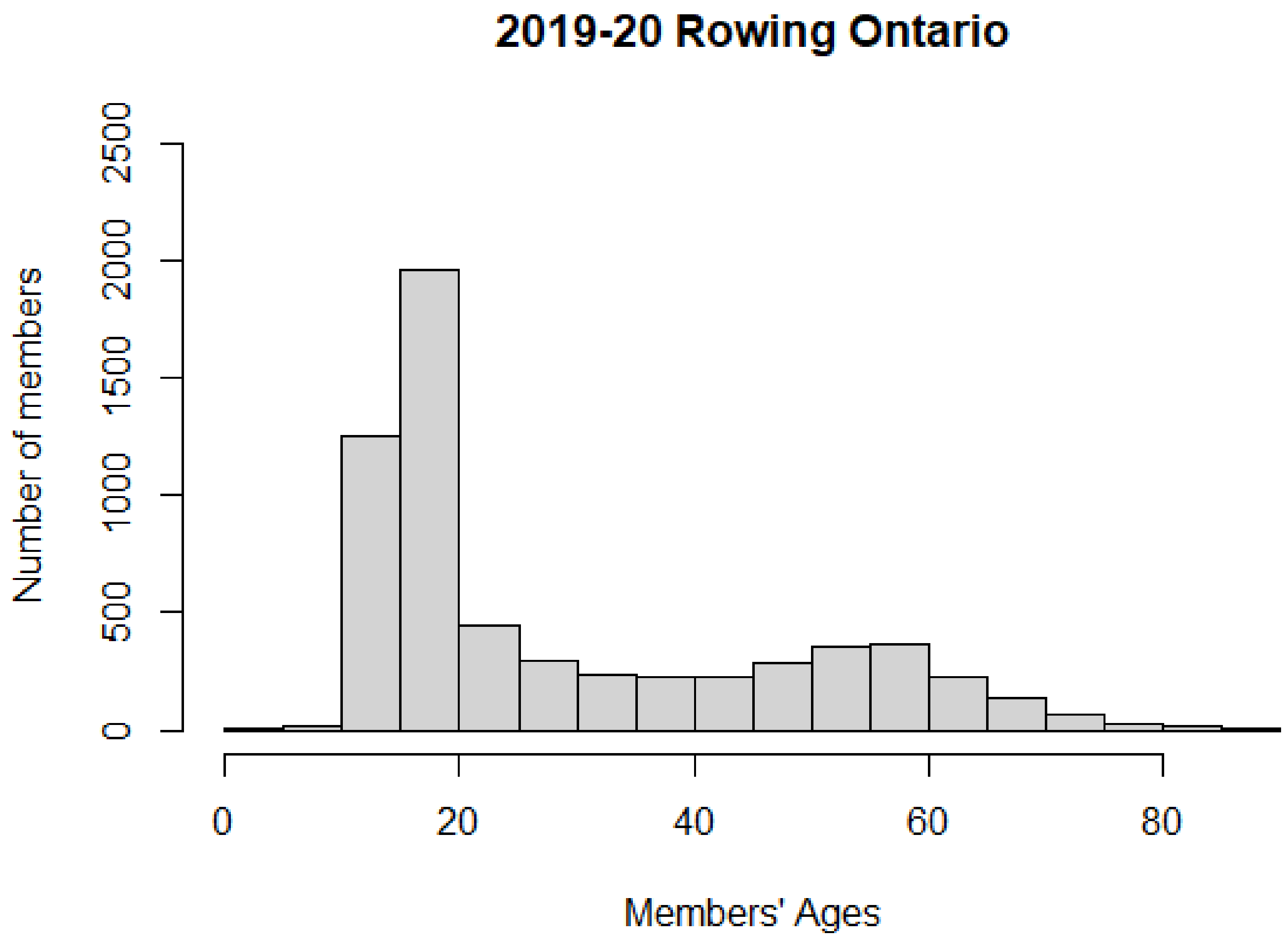

Age structure of all Row Ontario Members, 2019.

Functional Regions Identified by Row Ontario

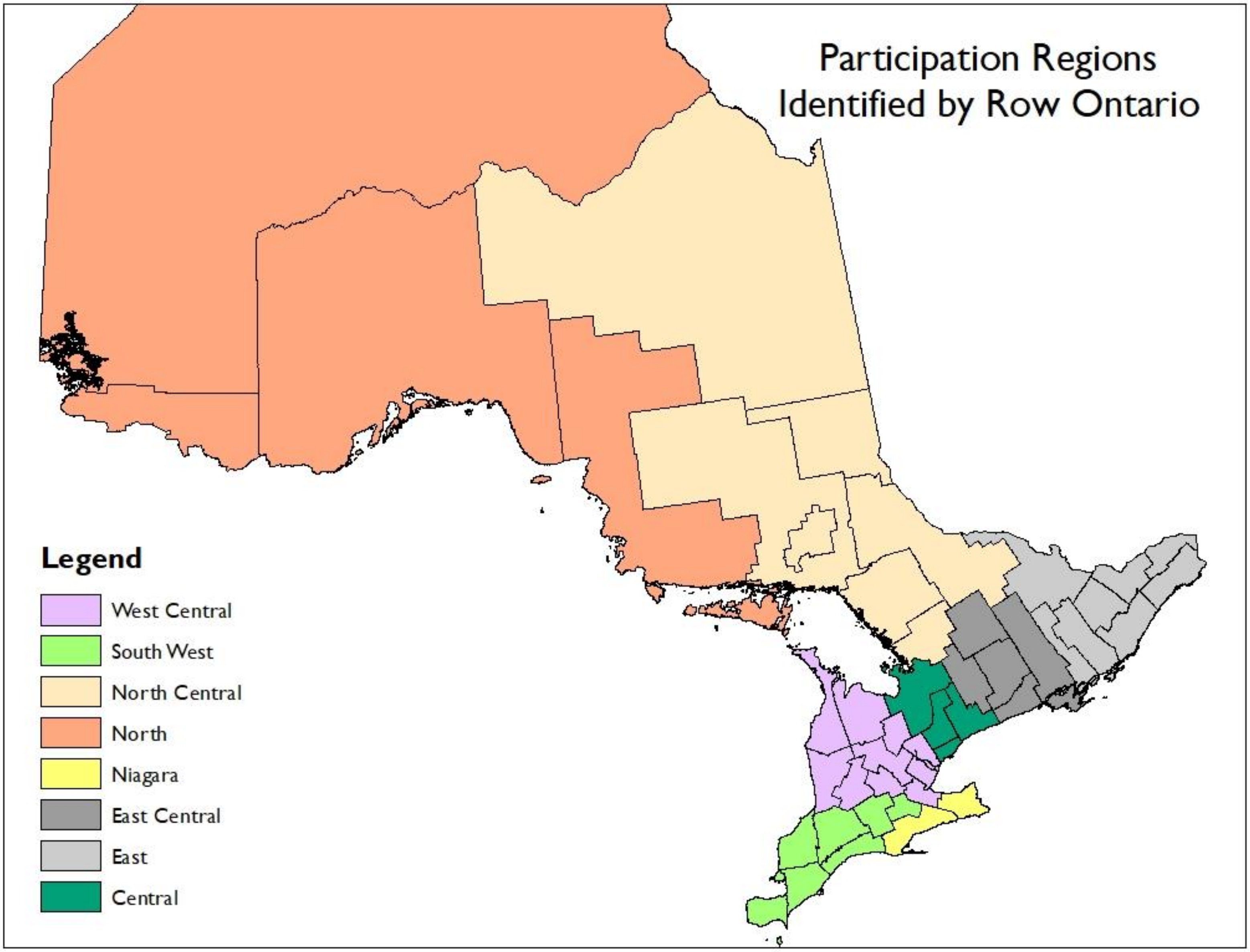

Rowing Clubs in the Central and West Central Regions

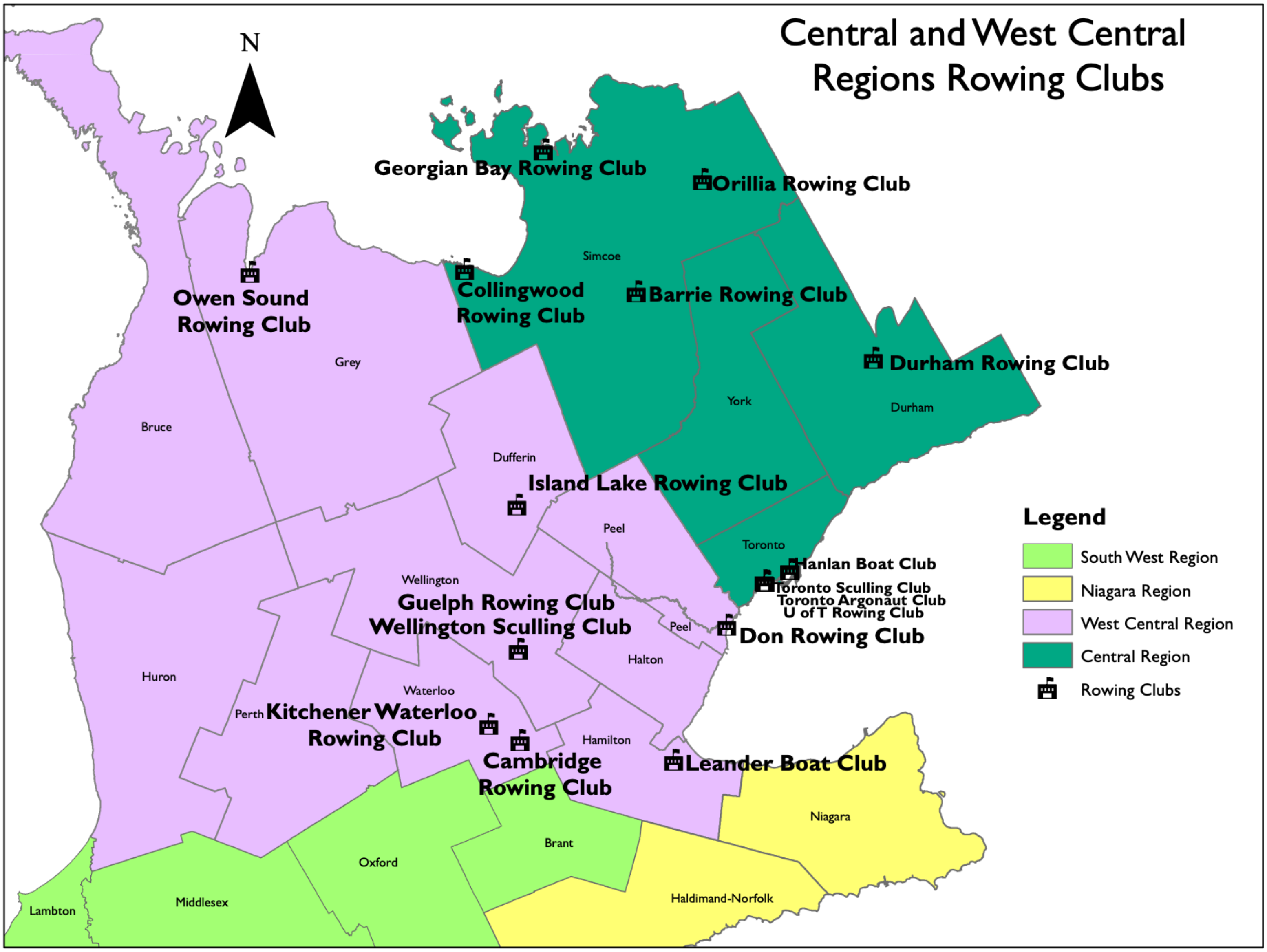

Number of Rowing Clubs on Ontario

	2014-15	2015-16	2016-17	2017-18	2018-19	2019-20
Number of clubs	51	51	52	51	50	53

Mean Age of All Participants by Region

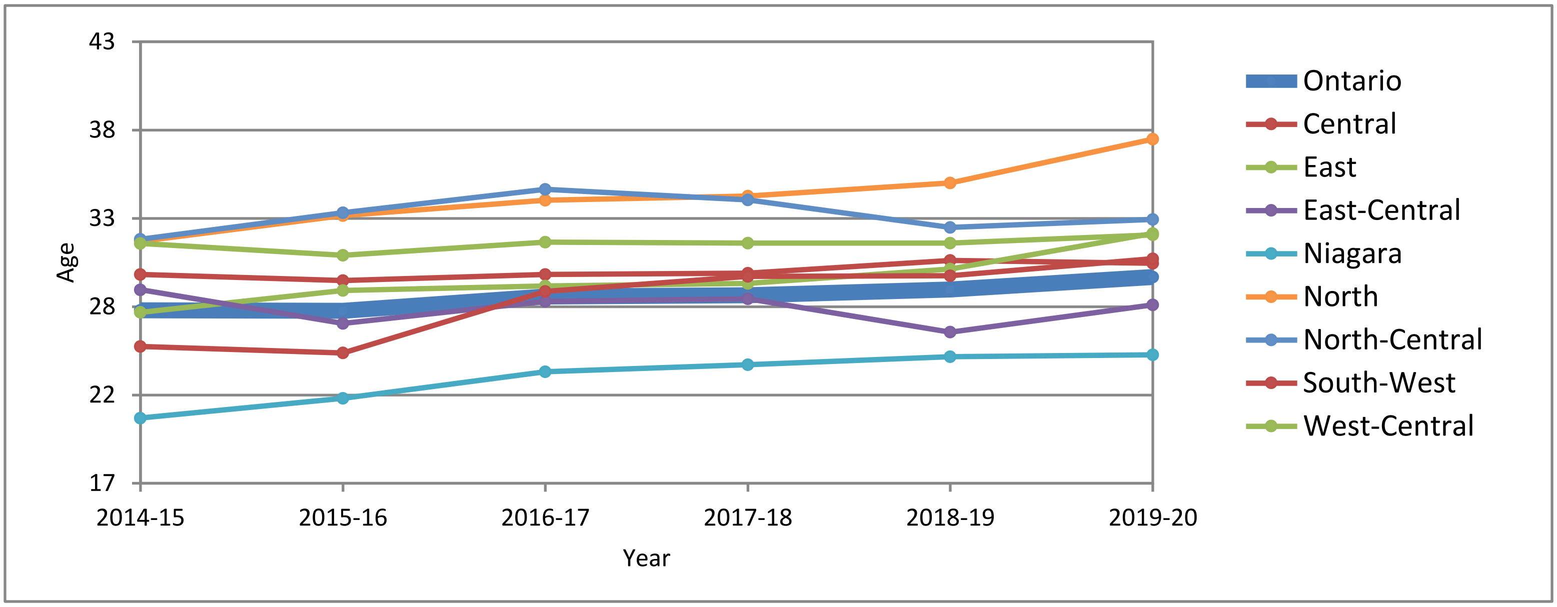

Mean Age of All Female Participants by Region

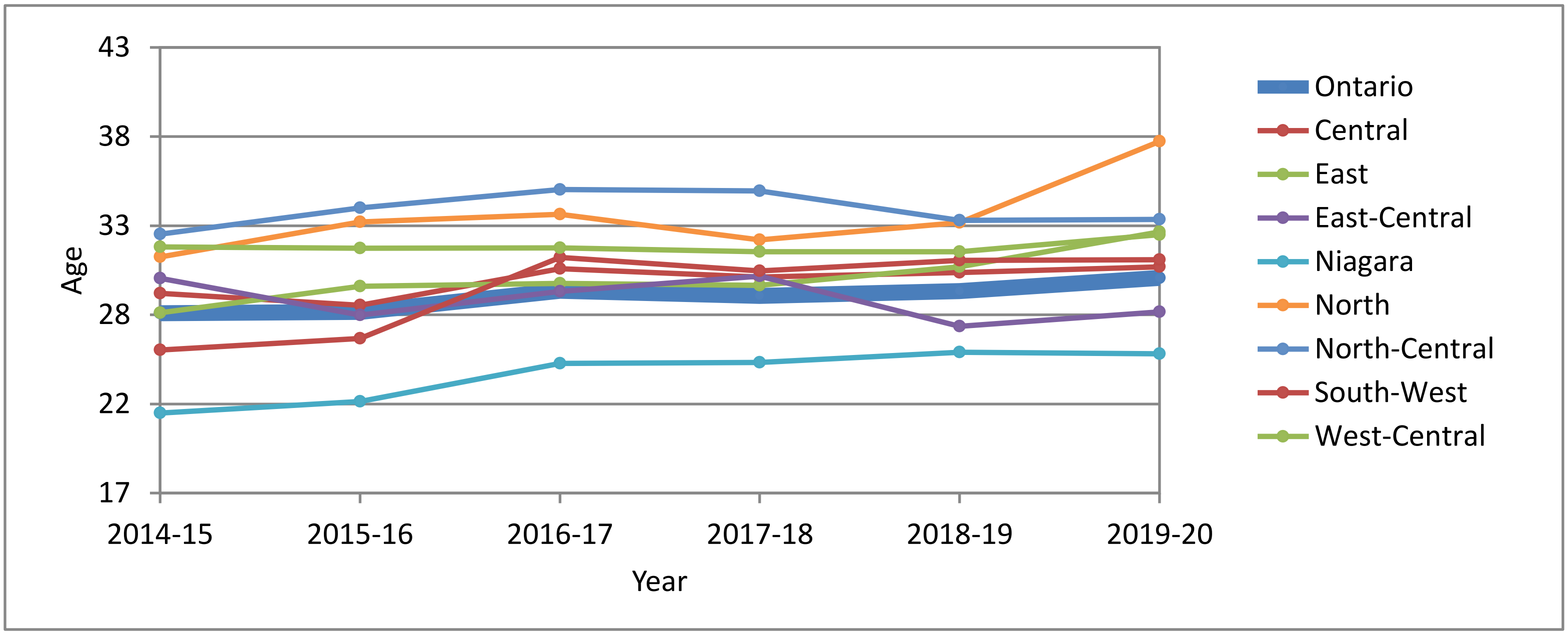

Average Number of Participants by Region (2014 – 2020)

Region	Average Number of Participants
Central	1466
East	1053
East-Central	367
Niagara	1689
North	77
North-Central	178
South-West	366
West-Central	1207

Total Participants by Region

	2014-15	2015-16	2016-17	2017-18	2018-19	2019-20
**Central**	1482	1502	1440	1321	1558	1490
**East**	934	1100	1649	996	900	738
**East-Central**	328	471	442	331	357	274
**Niagara**	1388	1620	1816	1783	1892	1635
**North**	88	50	66	70	80	105
**North-Central**	160	199	172	167	192	175
**South-West**	427	407	425	320	314	301
**West-Central**	1327	1309	1157	1063	1153	1235

Total Female Participants by Region

	2014-15	2015-16	2016-17	2017-18	2018-19	2019-20
**Central**	913	930	849	795	930	861
**East**	556	673	1003	607	519	449
**East-Central**	198	267	249	199	190	174
**Niagara**	813	933	1093	1083	1147	1018
**North**	53	29	38	43	55	73
**North-Central**	120	142	125	123	141	129
**South-West**	247	221	242	182	178	186
**West-Central**	792	772	695	610	691	748

Total Male Participants by Region

	2014-15	2015-16	2016-17	2017-18	2018-19	2019-20
**Central**	569	572	591	526	628	629
**East**	378	427	646	389	381	289
**East-Central**	130	204	193	132	167	100
**Niagara**	575	687	723	700	745	617
**North**	35	21	28	27	25	32
**North-Central**	40	57	47	44	51	46
**South-West**	180	186	183	138	136	115
**West-Central**	535	537	462	453	462	487

Mean Age All Participants by Region

	2014-15	2015-16	2016-17	2017-18	2018-19	2019-20
**Ontario**	27.3	27.3	28.1	28.2	28.5	29.3
**Central**	29.4	29.1	29.4	29.5	30.2	30.1
**East**	27.2	28.5	28.7	28.9	29.7	31.9
**East-Central**	28.5	26.5	27.8	28.0	26.0	27.6
**Niagara**	20.9	22.1	23.6	24.0	24.5	24.6
**North**	31.4	32.9	33.8	34.1	34.9	37.5
**North-Central**	31.5	33.1	34.5	33.8	32.2	32.7
**South-West**	25.1	24.7	28.4	29.3	29.3	30.4
**West-Central**	31.3	30.6	31.3	31.3	31.3	31.8

Mean Age Female Participants by Region

	2014-15	2015-16	2016-17	2017-18	2018-19	2019-20
Ontario	27.6	27.7	28.9	28.6	28.9	29.7
Central	28.8	28.1	30.2	29.7	30.0	30.3
East	27.6	29.2	29.4	29.2	30.3	32.4
East-Central	29.6	27.5	28.9	29.8	26.8	27.7
Niagara	21.7	22.4	24.6	24.7	25.3	25.2
North	30.9	33.0	33.4	31.9	32.9	37.7
North-Central	32.3	33.8	34.9	34.8	33.1	33.1
South-West	25.4	26.1	30.9	30.1	30.7	30.7
West-Central	31.5	31.4	31.4	31.2	31.2	32.2

Mean Age Male Participants by Region

	2014-15	2015-16	2016-17	2017-18	2018-19	2019-20
Ontario	26.8	26.6	26.9	27.5	28.0	28.6
Central	30.5	30.7	28.3	29.1	30.6	29.8
East	26.5	27.3	27.7	28.3	28.9	31.0
East-Central	26.8	25.2	26.5	25.3	25.0	27.5
Niagara	19.7	21.6	22.1	23.0	23.3	23.7
North	32.1	32.9	34.4	37.5	39.1	36.9
North-Central	29.3	31.3	33.4	31.2	29.8	31.5
South-West	24.7	23.1	25.1	28.2	27.5	29.7
West-Central	30.9	29.3	31.2	31.4	31.3	31.1
